# Differences in Plasma Cannabidiol Concentrations in Women and Men: A Randomized, Placebo-Controlled, Crossover Study

**DOI:** 10.3390/ijms241210273

**Published:** 2023-06-17

**Authors:** Ana Batinic, Davorka Sutlovic, Sendi Kuret, Franko Burcul, Nina Kalajzic, Antonela Matana, Goran Dujic, Josip Vrdoljak, Marko Kumric, Josko Bozic, Zeljko Dujic

**Affiliations:** 1 Pharmacy of Split-Dalmatia County, 21000 Split, Croatia; analiovic81@gmail.com; 2 Department of Health Studies, University of Split, 21000 Split, Croatia; sendikuret@gmail.com (S.K.); antonela.matana@gmail.com (A.M.); nkalajzic@ozs.unist.hr (N.K.); 3 Department of Toxicology and Pharmacogenetics, School of Medicine, University of Split, 21000 Split, Croatia; 4 Department of Analytical Chemistry, Faculty of Chemistry and Technology, University of Split, Ruđera Boškovića 35, 21000 Split, Croatia; franko@ktf-split.hr; 5 Clinical Department of Diagnostic and Interventional Radiology, University Hospital of Split, 21000 Split, Croatia; goran.dujic@gmail.com; 6 Department of Pathophysiology, School of Medicine, University of Split, 21000 Split, Croatia; josip.vrdoljak@mefst.hr (J.V.); marko.kumric@mefst.hr (M.K.); josko.bozic@mefst.hr (J.B.); 7 Department of Integrative Physiology, School of Medicine, University of Split, 21000 Split, Croatia; zeljko.dujic@mefst.hr

**Keywords:** DehydraTECH2.0 CBD, CBD metabolites, LC-MS analysis, SNP genotyping

## Abstract

The potential therapeutic benefits of cannabidiol (CBD) require further study. Here, we report a triple-blind (participant, investigator, and outcome assessor) placebo-controlled crossover study in which 62 hypertensive volunteers were randomly assigned to receive the recently developed DehydraTECH2.0 CBD formulation or a placebo. This is the first study to have been conducted using the DehydraTECH2.0 CBD formulation over a 12-week study duration. The new formulation’s long-term effects on CBD concentrations in plasma and urine, as well as its metabolites 7-hydroxy-CBD and 7-carboxy-CBD, were analyzed. The results of the plasma concentration ratio for CBD/7-OH-CBD in the third timepoint (after 5 weeks of use) were significantly higher than in the second timepoint (after 2.5 weeks of use; *p* = 0.043). In the same timepoints in the urine, a significantly higher concentration of 7-COOH-CBD was observed *p* < 0.001. Differences in CBD concentration were found between men and women. Plasma levels of CBD were still detectable 50 days after the last consumption of the CBD preparations. Significantly higher plasma CBD concentrations occurred in females compared to males, which was potentially related to greater adipose tissue. More research is needed to optimize CBD doses to consider the differential therapeutic benefits in men and women.

## 1. Introduction

A non-intoxicating and safely tolerated component of cannabis, cannabidiol (CBD), has been shown by numerous authors to have the ability to treat a variety of clinical conditions; therefore, therapeutic benefits of CBD in specialized populations continue to develop [1,2,3,4]. Previous studies have demonstrated that the oral bioavailability of CBD is very low [5,6]. Methods for accelerating the transport of ingested CBD to the bloodstream and preventing first-pass (hepatic) metabolism have been created through the use of inventive dietary supplements with various lipid formulations [7,8,9,10,11]. As an example, TurboCBD^TM^ (90 mg CBD; 1200 mg American ginseng; 480 mg ginkgo biloba; 300 mg organic hemp oil), which was consistent with higher peak CBD bioavailability, was associated with an increase in cerebral perfusion and a slight drop in blood pressure compared to baseline and the 90 mg control [8]. In their study, Kumric et al. found that serum catestatin and mean arterial pressure (MAP) levels were reduced after a 5-week treatment with DehydraTECH^TM^2.0 CBD formulation [10]. Dujic et al. found that in participants with untreated and treated hypertension, long-term CBD administration lowers ambulatory blood pressure [12]. In our prior research, only the DehydraTECH^TM^2.0 CBD formulation significantly decreased diastolic blood pressure and mean arterial pressure (MAP) in the first 20 min after ingestion of single dose, while both tested formulations (CBD and DehydraTECH^TM^2.0) led to a decrease in heart rate [13]. Since there were no serious adverse effects in either study, it was clear that the formulation was safe and acceptable [12,13]. We intended to expand on our previous study in which male subjects had more CBD in their urine than female subjects did [13]. There is still a huge lack of knowledge and understanding of sex-related differences in CBD therapy. Many researchers have explored how cannabis consumption differs depending on biological sex [14,15]. The research results are contradictory because some findings suggest that there is no significant sex difference in CBD concentrations in patient populations [16]. The findings of the study by Aviram et al. suggest that women are more at risk for medical-cannabis-related adverse events (AEs), probably due to the inherent sex effect [17]. Child and Tallon used an animal model of oral CBD consumption in their research and found that female rats consistently displayed higher levels of CBD in muscles and the liver. They have also found that this relationship was present in adipose tissue with low and medium CBD doses [18]. Maciel et al. reported that female mice had considerably higher CBD concentrations in their embryonic brains than males did [19]. These findings indicate tissue-specific pharmacokinetic interactions and higher plasma CBD concentrations in females than in males. Since there are a lack of human data, studies examining sex differences on the effects of CBD in clinical trials are required [20]. Genetic variants are linked to variations in population responses to CBD [21]. Cytochrome P450 (CYP) 2C19, CYP2C9, and CYP3A4 are mainly responsible for the metabolism of CBD [22,23,24,25,26]. In the current study, we analyzed the availability (i.e., circulating concentration) of the improved DehydraTECHTM2.0 CBD formulation in a larger number of participants with treated and untreated arterial hypertension (AH). This research was conducted based on our prior studies. The primary aim of this research was to examine the long-term effects of the new formulation on CBD concentrations as well as its metabolites [7-hydroxy-cannabidiol (7-OH-CBD) and 7-carboxy-cannabidol (7-COOH-CBD)]. Cannabinoid metabolism may be important for research because variations in the effects of cannabinoids may be connected to changes in the distribution of muscle mass and adipose tissue between men and women. Therefore, this research aimed to examine the relationship between the dose consumed and the concentrations achieved in relation to sex. The secondary goal of the study was to determine if there was a connection between CYP P450 enzyme polymorphism and the metabolism of DehydraTECH^TM^2.0 CBD formulation, as well as how long CBD and its metabolites remained in the body after the last dose.

## 2. Results

### 2.1. CBD and Metabolites Concentrations

Descriptive statistics (including minimum and maximum values, median, and interquartile range (IQR) for the CBD concentrations and metabolites, 7-OH-CBD and 7-COOH-CBD) are presented in Table 1. The baseline results for CBD concentrations and its metabolites showed a value of 0.0 for all individuals. Therefore, baseline data are not shown in further results.

Two timepoints (timepoints 2 and 3 for Group II and timepoints 5 and 6 for Group I) were utilized to compare the variations in CBD and CBD metabolite ratios and concentrations in plasma and urine using the Wilcoxon Signed Ranks Test. The results of the plasma concentration ratio for CBD/7-OH-CBD in the third timepoint (after 5 weeks of use) were significantly higher than in the second timepoint (after 2.5 weeks of use) *p* = 0.043. Likewise, the values of urine concentrations for 7-COOH-CBD in timepoint 3 were considerably higher than in timepoint 2, *p* < 0.001. Other variables did not show statistical significance.

In terms of sex, the concentrations of the metabolite 7-COOH-CBD in the urine sample at timepoint 3 were considerably higher in both women and men than they were at timepoint 2 (*p* = 0.027). The median at timepoint 2 was 2.92 ng/mL, while at timepoint 3 it was 9.06 ng/mL.

**Figure 1:**
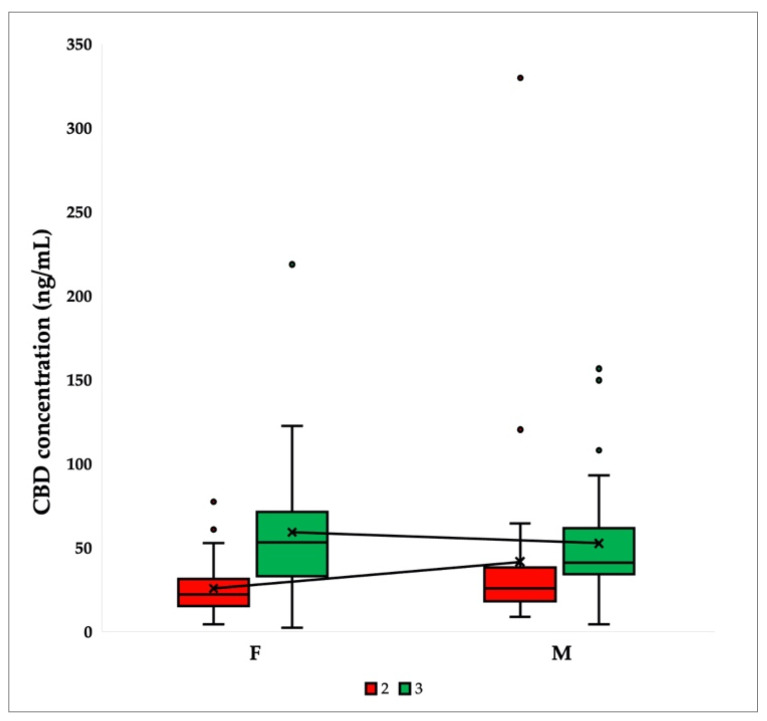
Comparison of CBD plasma concentrations at timepoints 2 (2.5 weeks after CBD ingestion) and 3 (5 weeks after CBD ingestion) stratified by sex (female—F and male—M). Box plots show the median, interquartile range (box), and total range (whiskers). Average values are connected by lines (N = 62).

Figure 1 shows sex differences in mean CBD plasma concentrations at timepoints 2 (after 2.5 weeks of consumption) and 3 (after 5 weeks of consumption). The results demonstrated a significant difference in median CBD concentration for timepoint 3. The median for women was 53,349 ng/mL, whereas the median for men was 41,171 ng/mL. At timepoint 2, there was no significant difference in the median CBD concentrations.

**Figure 2:**
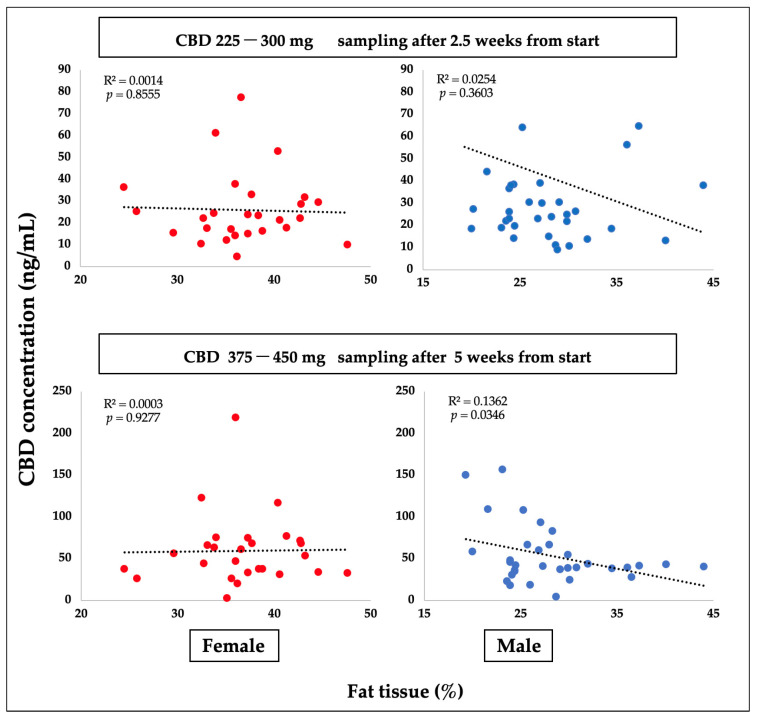
A graphical representation of the results of a linear regression analysis of pairs of dependent and independent variables: The concentration of CBD is represented by the dependent variable on the y axis, while the proportion of adipose tissue in female (F) and male (M) participants is represented by the independent variable on the x axis (N = 62).

CBD plasma concentrations were compared to body fat percentage in both sexes using linear regression analysis (Figure 2). For the male participants, a slightly negative trendline was found, but it was statistically significant only after 5 weeks (*p* = 0.0346).

The results for the concentrations of CBD and metabolites were zero at the baseline (timepoint 1 or timepoint 4, depending on whether CBD or placebo was taken first), with the exception of the group of participants who took CBD first and subsequently placebo (N = 31). Therefore, we aimed to establish whether CBD and/or its metabolites were present only in patients from Group II (who received CBD before placebo). The results are shown in Table 2. After the washout, the participants in Group II were examined, and the results revealed the presence of CBD and 7-OH-CBD metabolites in some of them (90.6% and 37.5%, respectively), while the presence of the 7-COOH-CBD metabolite was confirmed in all of the subjects. Following that, the participants took a placebo for 5 weeks, during which they went through testing twice (after 2.5 and 5 weeks). A smaller percentage of the participants were positive for the presence of CBD and 7-OH-CBD during both tests; however, all subjects remained positive for the presence of 7-COOH-CBD metabolites. In total, 50.0% of them were positive for the presence of CBD and 18.35% were positive for the presence of 7-OH-CBD after 2.5 weeks, while 15.6% and 9.4% were positive after 5 weeks.

**Figure 3:**
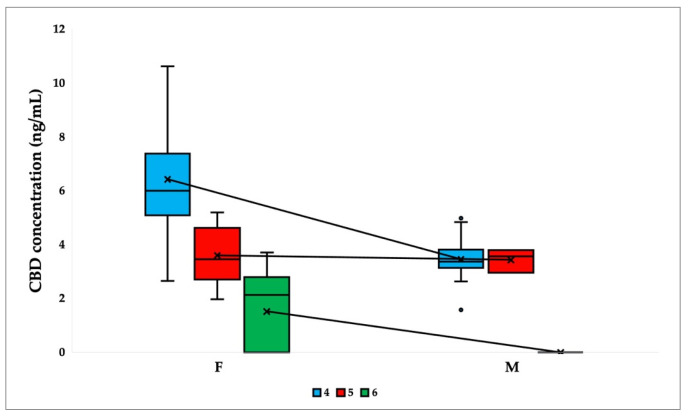
Comparison of CBD plasma concentrations at timepoints 4 (after washout), 5 (2.5 weeks after placebo capsule ingestion), and 6 (5 weeks after placebo capsule ingestion), stratified by sex (female—F and male—M). Box plots show the median, interquartile range (box), and total range (whiskers). Average values are connected by lines (N = 31).

The results revealed a significant difference in the median concentration of CBD in plasma at timepoints 4 (after washout) and 6 (5 weeks after placebo capsule ingestion) (Figure 3). The median for women at timepoint 4 was 5.994 ng/mL, while the median for men was 3.375 ng/mL. At timepoint 6, the CBD concentration in women was 2.13 ng/mL, but no men were CBD positive.

The concentration decreased in each timepoint that followed. Men and women had statistically different concentrations at practically all timepoints. In every instance where there was a statistically significant difference, the CBD or its metabolites’ plasma concentrations were higher in females compared to males.

**Figure 4:**
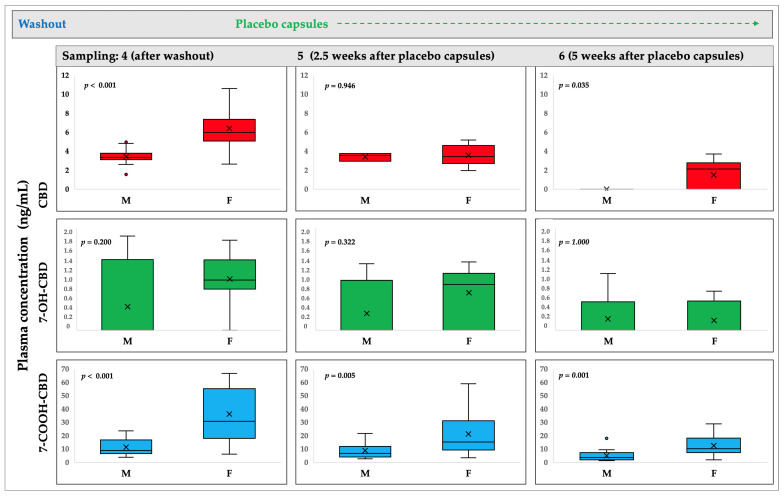
The Mann–Whitney test was used to compare the concentrations of CBD, 7-OH-CBD, and 7-COOH-CBD (ng/mL) in females (F) and males (M) at three different timepoints (4—after washout, 5—2.5 weeks after placebo capsules ingestion, and 6—5 weeks after placebo capsule ingestion). Box plots show the median, interquartile range (box), and total range (whiskers). Statistical significance is defined as *p* < 0.05.

Thus, at timepoints 4, 5, and 6, 16/17 (94%), 13/17 (76%), and 5/17 (29%) of the women tested positive for the presence of CBD. At the same timepoints, 12/14 (86%), 3/14 (21%), and 0/14 (0%) of the men were positive (Figure 4). The findings revealed a significant sex difference in CBD concentrations between timepoints 4 and 6 (*p* < 0.05). For any timepoint, there was no statistically significant difference in 7-OH-CBD concentrations, although there was a difference in 7-COOH-CBD concentrations across all timepoints.

**Figure 5:**
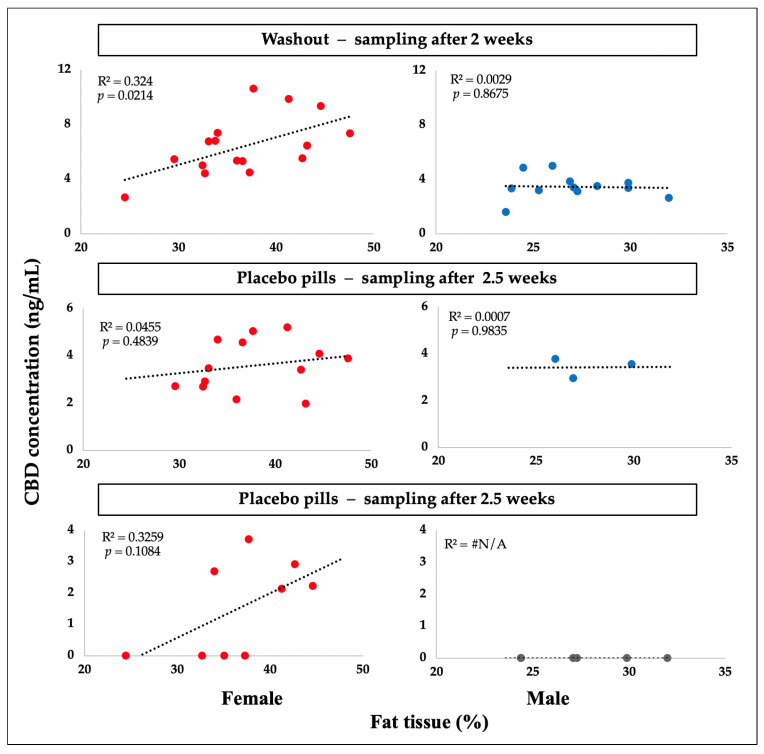
A graphical representation of the results of a linear regression analysis of pairs of dependent and independent variables: The concentration of CBD is represented by the dependent variable on the y axis, while the proportion of adipose tissue in female (F) and male (M) participants is represented by the independent variable on the x axis (N = 31).

Using linear regression analysis, plasma CBD concentrations were compared to body fat % in both sexes (Figure 5). The analysis only included patients who first consumed CBD and then placebo, and it refers to CBD concentrations in plasma after washout following placebo capsule intake. Female participants had a modestly positive trendline that was statistically significant after washout (*p* = 0.021). A slight positive correlation factor was found following the last testing, 5 weeks after the placebo capsules. No statistical significance was observed in men, but it should be noted that there were significantly fewer CBD-positive results in men at all timepoints indicated.

### 2.2. Association of Concentrations with CYP Genotype and Phenotype of Subjects

In total, 39% of the participants had a normal metabolism (NM) (genotype *1/*1), 56% had an intermediate metabolism (IM) (genotype *1/*2 or *1*3), and 5% had a poor metabolism (PM) (genotype *2/*2, *2/*3 or *3/*3), according to the CYP2C9*2*3 enzyme phenotype.

The CYP2C19*2*17 enzyme phenotype revealed that 39% of subjects had a normal metabolism (NM) (genotype *1/*1), 24% had an intermediate metabolism (IM) (genotype *1/*2 or *2/*17), 32% had a rapid metabolism (RM) (genotype *1/*17), 3% had an ultra-rapid metabolism (UR) (*17/*17), and 2% had a poor metabolism (PM) (genotype *2/*2).

Subjects were divided into categories for the CYP3A4 enzyme based on genotype. Consequently, 92% of them were wild type and 8% were heterozygous. No statistical significance was seen in the concentration differences at any timepoint when the CYP2C9 and CYP2C19 phenotypes were considered.

A statistically significant difference in the concentration of CBD in urine for timepoint 3 was seen for the CYP2C9 enzyme in male participants. Men with the NM phenotype had higher CBD values than males with the IM phenotype (*p* = 0.025). For any of the tested time intervals, the same enzyme in women showed no statistically significant changes. For any of the examined timepoints, no statistical difference was seen for the CYP2C19 enzyme in men. The level of 7-COOH-CBD in urine at timepoint 2 was shown to differ among women, though. The NM phenotype had the highest values, whereas the RM phenotype had the lowest values (*p* = 0.031).

At timepoint 2, the CBD/7-COOH-CBD concentration ratio had a statistically significant result for CYP3A4 (*p* = 0.037). In comparison to the wild type, the heterozygous genotype had a greater ratio of CBD/7-COOH-CBD.

## 3. Discussion

In this research, for the first time, steady-state CBD concentrations in plasma and urine samples were monitored over a 12-week period (84 days) with volunteers with hypertension. No statistical difference was observed in the CBD and CBD metabolites concentrations of participants who consumed and participants who did not consume angiotensin-converting enzyme (ACE) inhibitors, calcium blockers, and thiazide diuretics.

We found that the amount of CBD in the second 2.5 weeks of intake (5 weeks after the beginning of CBD consumption) increased by 50% compared to the first dose. CBD is mainly metabolized to 7-carboxy metabolites, as already confirmed by previous studies [2,27]. This final metabolite was found in plasma. When compared to the concentration after 2.5 weeks of CBD consumption, the metabolite’s concentration in plasma was nearly two times greater after 5 weeks of CBD use.

In contrast to our study, Perez-Acevedo et al.’s findings demonstrated that the concentration of 7-OH-metabolites in urine was higher than 7-COOH metabolites [28]. However, it should be noted that their study carried out measurements within 24 h, so the metabolite was not excreted to a great extent. This timeframe is in contrast to our study, in which measurements were carried out after 2.5 and 5 weeks from the start of CBD consumption, and with daily consumption. The plasma CBD/7-OH ratio decreased over time, which is in favor of an increase in metabolite concentration.

In their study, which lasted 7 days, Taylor et al. administered doses of 1500, 3000, 4500, and 6000 mg per day [2], which was significantly higher than in our study. The maximum concentrations of CBD and metabolites did not increase proportionally with increasing dose. Therefore, despite a four-fold increase in the dose (from 1500 mg to 6000 mg), the maximum concentrations were only 2.67 times higher. According to the findings of our study, only a 50% increase in CBD concentration was observed in our subjects’ plasma after 5 weeks of taking the supplement compared to the measurement taken after 2.5 weeks.

### 3.1. Sex Difference

By analyzing CBD plasma concentrations at the first measurement timepoint, after 2.5 weeks of CBD consumption, it was observed that CBD concentrations were higher in men than in women. As time progressed, and after repeated higher doses at the next timepoint (after 5 weeks), the concentration of CBD was higher in women than in men. Broadly consistent with these observations, in their rat study, Child and Tallon found that after 28 days of taking CBD, C_max_ increased by 36% in females, while it decreased by 22% in males [18].

The results of our study show that after stopping CBD intake and a two-week washout, CBD concentrations in female subjects remained significantly higher. More females than males tested positive for circulating CBD after the washout and 2.5 weeks of consuming the placebo capsules. Five weeks after taking the placebo, no men tested positively. In all subjects, at all examined timepoints, the presence of 7-COOH-CBD metabolites was significantly higher in women compared to men. Differences in concentration in favor of women may be due to their higher fat content compared to men. Due to its high lipophilicity, CBD disperses more readily in a lipophilic environment and is subsequently partly deposited, which affects cumulative concentration and slows elimination. According to Review by Millar et al. [29], C_max_ increased during the fed state and in lipid formulations, i.e., CBD can dissolve in the fat content of food, increasing its solubility and absorption, and consequently its bioavailability. Therefore, in order to promote optimal absorption, the same authors suggested that CBD should be administered orally after a meal.

Knaub et al. reported that healthy female volunteers may achieve higher CBD concentrations than healthy male volunteers, depending on the CBD formulation ingested [11]. Eight healthy female and eight healthy male participants were selected to receive a single dose of 25 mg of CBD orally in either a novel self-emulsifying drug delivery system formulation (SEDDS-CBD) or a medium-chain triglycerides formulation (MCT-CBD). Females had considerably greater area under curve (AUC) for the MCT-CBD formulation than males (for comparison, DehydraTECH™2.0 CBD has long-chain fatty acids associated with the CBD); however, for the SEDDS-CBD formulation, males had a more rapid t_max_ [11]. In our study, the CBD/7-OH-CBD ratio was higher in males than in women at 2.5 weeks following CBD consumption (1.97 vs. 1.68), but it was nearly equal after 5 weeks (1.89 vs. 1.87). It could be concluded that in the initial measurement, males had more CBD in their plasma than women, possibly because some of the CBD in women had already been stored in adipose tissue. Seeboth et al. [30], in their study involving 16 women and 27 men, analyzed the pharmacokinetic variations in a single oral dose of 15 mg of THC. Their results for maximum concentrations and average area under the curve showed differences between women and men. Consistent with the current findings, in women, these values were about 1.5 times higher than in men. These differences were not related to genotype or phenotype differences, but to differences in the volume of distribution, which is influenced by differences in the percentage of body fat.

A statistical correlation was observed with the percentage of body fat in the subjects. After 5 weeks of CBD ingestion, male individuals had a negative correlation, while females after washout had a positive correlation. According to the results of our study, the values of body fat percentage of female subjects were significantly higher compared to men, while at the same time, the percentages of muscle tissue and water were significantly lower. This likely explains the difference in the higher plasma CBD concentrations achieved at the later measurement timepoints. The findings of the study by Child and Tallon, which was carried out on rats (groups of six males and six females) over the course of 28 days, demonstrated that CBD accumulated in the tissues that were examined, including fat, muscle, and the liver, with a suggestion that higher values were found in adipose tissue and in female rats [18].

There has been a lot of research in recent years on the pharmacokinetics and pharmacodynamics of CBD and the differences between the sexes, but the studies were either acute, did not involve many subjects of either sex, or were not conducted on humans [14,18].

Our findings revealed that the presence of CBD in the plasma was evident in female participants 50 days after the last intake of CBD preparations. The difference in fat tissue between men and women is likely the reason for such findings. The fat tissue content of our study participants differed significantly in favor of women. It was 74% higher. Child and Tallon’s research on rats of both sexes, showed significant changes in concentrations related to the time of the measurements [18]. CBD was administered to rats every day for 28 days. C_max_ was similar in both sexes, and on the first day, it was 3.7% higher in female rats. On the 28th day, female rats compared to male rats had a 66% higher concentration. The variables controlling the metabolism and tissue accumulation of ingested CBD are complex. Fatty acid binding proteins are important in the intracellular transport of CBD [31]. Albumin is the major transporter of CBD in the extracellular compartment; 90% of CBD is linked to proteins. Women, compared to men, have a lower mean concentration of albumin in their serum at the age of 20–60 years [32]. Such information can be used to personalize oral CBD dosing and maximize therapeutic doses in women.

In our previous study [13], in the 180th minute after ingestion, significantly higher concentrations of CBD in urine were found in male subjects than in female subjects. The results obtained are in accordance with the results of this study. In male subjects, due to a lower percentage of fat tissue, a lower accumulation of CBD was achieved, and consequently, a higher concentration of CBD remained in the bloodstream. Most of it from the bloodstream was metabolized and excreted in the urine, i.e., it was eliminated from the body more quickly. Also, it is stated [32] that the level of albumin may be responsible for the achieved concentrations of CBD. Perhaps the larger albumin content accelerated metabolism and, as a result, excretion, resulting in a lower plasma CBD concentration.

### 3.2. Implications

A positive screening test for the presence of cannabinoids long after the cessation of consumption should also be noted as information on CBD products and should serve as a caution to all future users. In particular, it is well known that, in the majority of nations, if a person’s test is positive for the presence of cannabinoids, they are not permitted to operate machines or a motor vehicles [33,34]. We also observe that in certain situations, testing is only conducted using quick and insufficiently precise screening tests. Since this relates to CBD or its inactive metabolites, 7-OH-CBD and 7-COOH-CBD, it is essential to mention this in the consumption instructions so that if users are tested with rapid screening tests and are still positive, they know to insist on confirmatory tests before possible sanctions.

### 3.3. CYP Genotype and Phenotype

Our results showed that there was no association of CBD concentrations of concentration-matched CBD formulation with any phenotype or genotype. An exception was observed only for the concentration of CBD in men after 5 weeks of consumption, and related to the CYP2C9 enzyme phenotype. Men with the NM phenotype had significantly higher values of CBD in urine compared to subjects with IM phenotype. Also, a difference in the CYP2C19 enzyme phenotype was observed among female subjects. Although this was compared to subjects with the RM phenotype, subjects with the NM phenotype had greater values here as well. Perhaps this is the result of the formulation used, which bypasses the first pass through the liver, where the tested enzymes are located. There are also studies that have shown that cannabinoids and their metabolites can inhibit some P450 enzymes [35], which could be a basis for further discussion and additional research. It should be mentioned that some studies have demonstrated that healthy individuals who ingested doses of CBD of 1500 mg/day revealed an increase in serum alanine aminotransferase (ALT) values, which correlate with drug-induced liver damage. In the current study using several-fold-lower CBD dosage serum, liver enzymes were not affected [36].

### 3.4. Study Limitations

A limitation of the study is that serum albumin levels were not measured for all participants. Hormonal status was not included to better understand the difference between the sexes.

## 4. Materials and Methods

### 4.1. Participants and Ethical Approval

For this study, we selected 62 hypertensive participants (27 women and 35 men) aged between 40 and 70 years. The research was carried out in accordance with the Helsinki Declaration. Before being included in the trial, all participants provided written informed permission, and the trial was approved by the Ethics Committee of the Faculty of Medicine at the University of Split on 15 December 2021 (Class: 003-08/21-03/0003; Registration number: 2181-198-03-04-21-0091). The HYPER-H21-4 trial is registered on ClinicalTrials.gov as NCT05346562. The study’s inclusion and exclusion criteria were described in our prior article [9].

### 4.2. Anthropometrics and Background

The Tanita scale (DC-360 S; Tanita, Tokyo, Japan) was used to measure weight, metabolic age, visceral fat, and the proportions of fat, muscle tissue, and water. Body mass index was calculated as weight divided by squared height. Results are shown in Table 3.

Of the total number of participants, 14 were taking angiotensin-converting enzyme (ACE) inhibitors, 13 were taking ACE inhibitors and calcium blockers, and three were taking ACE inhibitors and thiazide diuretics.

### 4.3. Research Design

**Figure 6:**
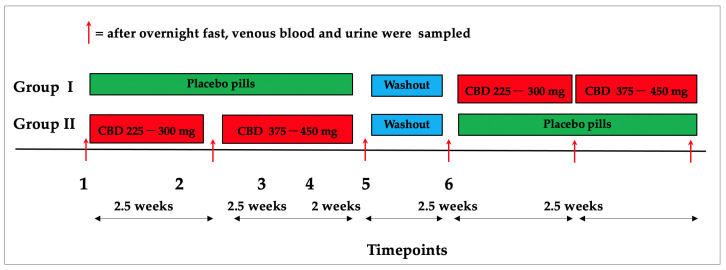
Research design. DehydraTECH™2.0 CBD (225 to 300 mg split over three times daily for the initial 2.5 weeks and 375 to 450 mg split over three times for the following 2.5 weeks). Participants repeated tests for a further five weeks under varied circumstances after a two-week washout. Three times in each study arm, participants visited the laboratory on a total of six occasions, each after an overnight fast.

The participants were randomly divided into two groups (Figure 6). For five weeks, participants randomized to the first group (placebo, then cannabidiol) received placebo capsule matching a cannabidiol one. After a two-week washout period, participants received cannabidiol in the following doses: 225 to 300 mg (depending on the sex and weight of participants) divided three times per day for the first 2.5 weeks, then 375 to 450 mg (depending on the sex and weight of participants) divided three times for the next 2.5 weeks. Participants in the second group (cannabidiol, then placebo) received cannabidiol in the amounts of 225 to 300 mg divided three times daily for the first 2.5 weeks and 375 to 450 mg divided three times daily for the next 2.5 weeks. Participants received cannabidiol-matched placebo capsules for the next five weeks after a two-week washout period. For blinding reasons, the DehydraTECH™2.0 CBD formulation and the placebo substrate powder were packed into capsules of comparable dimensions and shape. A detailed description of the formulation, intake, and overall regimen of the study was described in a previous paper [9].

### 4.4. Sample Collection and Storage

After a 12 h fast, venous blood samples were collected from the antecubital vein in each individual. Plasma was separated (centrifuged at 4 °C on 3500 rpm for 10 min) and kept at −20 °C within 1–2 h of collection. Each sample was marked with the study number, the test session number, the date, and the time for each patient. Per patient, two urine samples (5–10 mL) and three plasma samples (1.5–2 mL) were sent directly to the lab for evaluation. All plasma and urine samples were tested in the same laboratory in accordance with proper laboratory practices, and analysts were unaware of the distribution of patients in the studied groups.

### 4.5. Plasma and Urine Samples Extraction

Proteins in plasma samples (1 mL aliquots) were precipitated with 1.25 mL of ice-cold acetonitrile. After mixing, samples were centrifuged (2600 rpm for 2 min) and 1.5 mL of supernatant with 1 mL d.d. H_2_O, as well as urine sample, was added to preconditioned solid phase extraction (SPE) columns with CBD specific cartridges (United Chemical Technologies, Styre Screen SSTHC063, Bristol, PA, USA; for both, plasma and urine extraction protocol were carried out according to the manufacturer’s instructions, Bristol, PA, USA). Subsequently, the column was rinsed with 1 mL dd H_2_O and dried under high vacuum (~20 inch). CBD was eluted with a 3 mL mixture of hexane/ethyl acetate/acetic acid (49:49:2, *v*/*v*) and dried under nitrogen. Samples were reconstituted with 150 μL mix of acetonitrile/d.d. H_2_O (1:1, *v*/*v*).

### 4.6. Standard Solutions

Standards used for qualitative and quantitative determination of cannabidiol (CBD) and its metabolites 7-hydroxy-cannabidiol (7-OH-CBD) and 7-carboxy-cannabidol (7-COOH-CBD) were purchased from Cerilliant, Sigma Aldrich (Round Rock, TX, USA), as certified reference solutions in methanol 1 mg/mL each (Product codes: C-045-1ML, C-180-1ML and C-181-1ML, respectively).

### 4.7. LC-MS Analysis

Measurements were performed on the UHPLC-MS/MS (Ultimate 3000RS equipped with TSQ Quantis MS/MS detector, Thermo Fischer Scientific, Waltham, MA, USA). Heated electrospray ion source (H-ESI) was set to a positive ion mode with spray voltage of 4000 V, while Sheat, Aux, and Sweep gasses were set to 50, 25, and 2 arbitrary units (Arb), respectively. Ion transfer tube was set to 325 °C and Vaporizer temperature was set to 280 °C. Collision-induced dissociation (CID) gas pressure was constant at 1.5 mTorr. Sample injection volume was 1 μL. Separation was performed on Accucore C18 column (150 mm × 2.1 mm, 2.6 µm, Thermo Fischer Scientific, Waltham, MA, USA) equipped with Accucore C18 guard column (10 mm × 2.1 mm, 2.6 µm) using gradient elution with 0.1% formic acid in water (solvent A) and 0.1% formic acid in acetonitrile (solvent B). Solvent gradient was programmed as follows: 0 min 60% B, 8 min 68% B, 8.1 min 95% B, 9.1 min 95% B, 9.3 min 60% B, and 11 min 60% B. Total run time was 11 min. Column flow was 0.5 mL/min. Column temperature was held at 40 °C, while autosampler temperature was held at 15 °C. Needle was washed in solvent mixture comprised of acetonitrile/methanol/water/formic acid = 40:40:20:2 (*v*/*v*).

Quantitative analysis of CBD, 7-OH-CBD, and 7-COOH-CBD was performed using external calibration curves for each analyte (ranging from 0 ng/mL to 2000 ng/mL). Each compound was introduced to the MS/MS system separately to optimize fragment transitions, collision energies, and ion focusing lens voltages, which were all performed using Chromeleon MS tuning console v. 7.2.10 (23925) and are shown in the following Table 4.

Limit of detection (LOD) and limit of quantification (LOQ) were estimated using Blank Samples approach. Ten (10) blank samples were analyzed and LOD was calculated as 3.9*(s_y,bl_/b), where s_y,bl_ represents the standard deviation of the blank signal for each analyte and b represents the slope of the appropriate calibration curve. LOQ was estimated as 3.3*LOD. Both LOD and LOQ and the appropriate calibration curve equations are shown in the following Table 5.

### 4.8. DNA Analysis and SNP Genotyping

From the baseline and regular check-ups, blood samples for DNA research were taken and kept in EDTA tubes. DNA isolation, DNA quantification, and SNP determination were performed according to our previous study [13].

### 4.9. Statistical Analysis

The Kolmogorov–Smirnov test was used for normality checking. Due to the non-normal distribution of the data, continuous variables are presented with the minimum, maximum, and median (interquartile range, IQR), and categorical variables are presented with frequencies (percentages). Groups were compared using the non-parametric Mann–Whitney U test, linear regression analysis, and Wilcoxon Signed Ranks test. *p*-values of less than 0.05 were considered statistically significant. Furthermore, multivariate linear regression analysis was performed to assess the association of CYP genotypes with CBD levels in the plasma and urine. Statistical analysis was performed using Statistical Package Software for Social Science, version 28 (SPSS Inc., Chicago, IL, USA).

## 5. Conclusions

CBD’s effect is affected by its bioavailability, and larger concentrations of CBD in plasma or serum correlate well with it. In order for a person to achieve a higher concentration, it is not necessary to exclusively take more daily doses. We assume that a longer period of intake has a greater impact on more effective concentrations than a higher daily dose. Over a longer period of time, women achieve a higher plasma concentration of CBD.

CBD plasma concentrations in men were negatively correlated with their amount of adipose tissue. It can be assumed that the rate constant of absorption and elimination of CBD in men compared to women is higher, which may mean faster metabolism of CBD in men and consequently lower CBD concentrations.

To examine the concentrations and effects of CBD over a period of several months, we believe that further research is needed. We found no studies in the literature relating to differences in CBD concentrations in participants based on sex hormone status. The majority of the test subjects in our study were postmenopausal. We believe that this parameter should be researched further as well. Additionally, it would be beneficial to look into the effects of combining CBD with medications that are more frequently used, such as those that decrease pain, fever, or other symptoms.

## Figures and Tables

**Table 1:** Descriptive statistics for the CBD concentrations and metabolites 7-OH-CBD and 7-COOH-CBD in plasma and urine samples and plasma ratio. The findings only apply to samples taken after ingesting CBD at two different timepoints after 2.5 and 5 weeks. Timepoints 5 and 6 for Group I and timepoints 2 and 3 for Group II. In the following text, we will refer to them as timepoint 2 (2 or 5—after 2.5 weeks) or timepoint 3 (3 or 6—after 5 weeks). N = 62.

Variable		Timepoint 2 (after 2.5 weeks)			Timepoint 3 (after 5 weeks)		
		Min	Max	Median (IQR)	Min	Max	Median (IQR)
Plasma concentration (ng/mL)	CBD	10.53	329.88	23.81 (20.36)	18.36	149.97	47.1 (35.53)
	7-OH-CBD	2.72	127.23	13.15 (13.90)	6.40	109.93	30.01 (28.19)
	7-COOH-CBD	195.79	5415.25	837.15 (883.48)	575.72	9300.95	1589.77 (2786.08)
Plasma ratio	CBD/−7OH-CBD	0.62	5.67	1.96 (1.30)	0.62	6.19	1.84 (1.87)
	CBD/7-COOH-CBD	0.005	0.20	0.03 (0.02)	0.007	0.12	0.03 (0.03)
	7OH-CBD /7COOH-CBD	0.006	0.08	0.02 (0.007)	0.007	0.03	0.01 (0.008)
Urine concentration (ng/mL)	CBD	0	9.93	0 (0)	0	6,59	0 (0)
	7-OH-CBD	0	81.86	0 (11.074)	0	66,35	0 (7.43)
	7-COOH-CBD	0	43.87	2.92 (5.68)	0	265,15	9.06 (9.813)

Abbreviations: CBD, cannabidiol; 7-OH-CBD, 7-hydroxy-CBD; 7-COOH-CBD, 7-carboxy-CBD; IQR, interquartile range.

**Table 2:** Descriptive statistics for the CBD concentration and metabolites 7-OH-CBD and 7-COOH-CBD in plasma and urine samples at timepoints 4–6 (2.5 and 5 weeks after placebo capsules ingestion). The results refer only to Group II participants (N = 31) for which samples were collected while taking placebo capsules.

Variable		Timepoint 4 (after Washout)			After Placebo Capsule Ingestion					
					Timepoint 5 (after 2.5 Weeks)			Timepoint 6 (after 5 Weeks)		
		Min	Max	Median (IQR)	Min	Max	Median (IQR)	Min	Max	Median (IQR)
Plasma concentration (ng/mL)	CBD	1.58	10.63	4.91 (3.15)	1.97	5.2	3.51 (1.34)	0	3.71	0 (2.17)
	7-OH-CBD	0	1.85	0.91 (0.78)	0	1.34	0.77 (1.07)	0	1.109	0 (0)
	7-COOH-CBD	3.87	66.85	18.77 (24.28)	2.81	59.11	11.19 (16.05)	1.42	28.94	7.36 (8.93)
Urine concentration (ng/mL)	CBD	0.0	0.0	0.0 (0.0)	0.0	0.0	0.0 (0.0)	0.0	0.0	0.0 (0.0)
	7-OH-CBD	0.0	1.20	0.0 (0.0)	0.0	5.11	0.0 (0.0)	0.0	0.93	0.0 (0.0)
	7-COOH-CBD	0.0	73.81	0.0 (0.69)	0.0	316.24	0.0 (0.41)	0.0	8.17	0.0 (0.0)

Abbreviations: CBD, cannabidiol; 7-OH-CBD, 7-hydroxy-CBD; 7-COOH-CBD, 7-carboxy-CBD; IQR, interquartile range.

**Table 3:** Antropometric summary and descriptive statistics of participants.

Variable		Min	Max	Median (IQR)	Sex Difference Mann–Whitney Test
Age	F	43	70	55 (12)	*p* = 0.230
	M	42	69	54 (12)	
Weight	F	62.00	101.70	78.30 (15.10)	*p* < 0.001
	M	79.30	126.80	95.90 (14.30)	
Body Mass index (BMI)	F	20.88	33.87	28.43 (4.96)	*p* = 0.659
	M	24.55	34.97	28.17 (5.08)	
Fat tissue (%)	F	24.50	47.60	36.60 (6.80)	*p* < 0.001
	M	19.30	44.00	27.10 (6.20)	
Muscle tissue (%)	F	49.70	71.60	60.10 (6.40)	*p* < 0.001
	M	53.30	76.60	69.30 (5.60)	
Body water (%)	F	37.00	52.50	42.20 (4.60)	*p* < 0.001
	M	41.00	54.90	49.40 (3.50)	
Metabolic age (years)	F	35.00	77.00	58.00 (14.00)	*p* = 0.795
	M	40.00	82.00	60.00 (13.00)	
Fat free mass (kg)	F	42.30	59.40	51.10 (8.10)	*p* < 0.001
	M	60.00	89.00	70.50 (8.80)	
Visceral fat (kg)	F	4.00	14.00	9.00 (4.00)	*p* < 0.001
	M	8.00	31.00	12.00 (4.00)	

Data presented as minimum, maximum, median, and interquartile range (IQR).

**Table 4:** Quantitative and qualitative transitions used for analysis of CBD and its metabolites.

Compound	Q1 (m/z)	Q3 ^a^ (m/z)	Q3 ^b^ (m/z)	RF (V)	CE (eV)	CE (eV)	RT (min)
CBD	315.5	193.1	259.2	115	21	18	5.57
7-OH-CBD	313.5	201.1	193.2	134	22	22	1.48
7-COOH-CBD	345.5	327.2	299.3	109	14	18	1.33

Abbreviations: CBD—cannabidiol; 7-OH-CBD—7-hydroxy-CBD; 7-COOH-CBD—7-carboxy-CBD; Q1—precursor ion; Q3—product ion (a—quantifying, b—qualifying); RF—focusing lens voltage; CE—collision energy; RT—retention time.

**Table 5:** Quantitative and qualitative transitions used for analysis of CBD and its metabolites.

Compound	LOD (ng/mL)	LOQ (ng/mL)	Calibration Curve Equation	R^2^
CBD	0.60	1.98	y = 3.1186x	0.995
7-OH-CBD	0.20	0.66	y = 2.7706x	0.998
7-COOH-CBD	0.20	0.66	y = 9.6546x	0.998

Abbreviations: CBD—cannabidiol; 7-OH-CBD—7-hydroxy-CBD; 7-COOH-CBD—7-carboxy-CBD; LOD—limit of detection; LOQ—limit of quantification; R^2^—correlation coefficient.

## Data Availability

The data presented in this study are available on request from the corresponding author.
